# Adaptive graph learning of microbial phylogeny enables accurate and interpretable microbiome-based host phenotype prediction

**DOI:** 10.1128/aem.00788-26

**Published:** 2026-07-10

**Authors:** Biao Dong, Bin Wang, Jiongjin Chen, Xiaomin Xu, Zhenjiang Zech Xu

**Affiliations:** 1 State Key Laboratory of Food Science and Resources, Nanchang University 47861, Nanchang, China; 2 School of Mathematics and Computer Sciences, Nanchang University 47861, Nanchang, China; 3 Shenzhen Hospital, Southern Medical University, Shenzhen, Guangdong, China; University of Illinois Urbana-Champaign, Urbana, Illinois, USA

**Keywords:** gut microbiome, phylogeny, graph neural network, edge embeddings, interpretability, biomarker discovery

## Abstract

The human microbiome is inherently structured by phylogeny, yet most predictive models treat microbial taxa as independent features, thereby underusing evolutionary information that may improve disease classification. While recent deep learning approaches have attempted to incorporate phylogeny, they generally rely on projecting phylogenetic trees into Euclidean spaces, which can distort the intrinsic topology of evolutionary relationships. To address this limitation, we propose PhyloGCNE, a framework that models microbiome samples directly as graphs and employs edge-aware graph convolution to integrate phylogeny. Unlike previous methods that rely on fixed, distance-based aggregation, PhyloGCNE learns how phylogeny-informed edge attributes should influence signal propagation across evolutionary hierarchies. We further introduce a Phylogenetic Saliency Propagation (PSP) framework for model interpretation, which attributes importance scores to microbial taxa by integrating gradient sensitivity with evolutionary context. Benchmarked against one synthetic and eight real-world data sets spanning inflammatory bowel disease, colorectal cancer, type 2 diabetes, oral squamous cell carcinoma, gastric cancer, and dietary fiber intervention, PhyloGCNE consistently outperforms existing state-of-the-art approaches. Together, these results establish PhyloGCNE as an accurate and interpretable phylogeny-aware framework for microbiome-based host phenotype prediction.

**IMPORTANCE:**

The human microbiome is a complex ecosystem closely linked to physiological health, yet traditional analysis often treats microbes as isolated features, ignoring their shared evolutionary history. This study introduces PhyloGCNE, a novel framework that integrates the evolutionary tree directly into the analysis of microbiome data. By modeling microbial communities as interconnected networks rather than independent entities, this approach captures shared biological traits across related lineages. We demonstrate that this method significantly improves the accuracy of predicting host phenotypes, such as inflammatory bowel disease and colorectal cancer. Crucially, unlike many “black box” artificial intelligence models, this tool identifies specific, biologically relevant microbial signatures driving these predictions. This advancement provides a powerful, interpretable approach for deciphering the complex links between the human microbiome and host phenotypes.

## INTRODUCTION

The human microbiome constitutes a complex ecosystem that serves as a critical indicator of host physiological states. Significant structural shifts in this community are observed not only in the context of severe pathologies, such as inflammatory bowel disease (IBD) (1), colorectal cancer (CRC) (2), and type 2 diabetes (T2D) (3), but also in response to therapeutic or dietary interventions, such as dietary fiber supplementation (4), reflecting a broader One Health perspective that links microbiome dynamics to lifestyle, nutrition, and environmental factors (5). With the widespread adoption of high-throughput sequencing technologies, such as 16S rRNA amplicon sequencing and whole-genome shotgun (WGS) sequencing, the rapid growth of microbiome data has provided unprecedented opportunities for data-driven host phenotype identification and biomarker discovery. Consequently, leveraging machine learning (ML) algorithms to decipher the complex interplay between microbial communities and host phenotypes has emerged as a cornerstone of both precision medicine and personalized nutrition.

However, applying traditional ML methods directly to microbiome data presents substantial challenges (6). First, microbiome data are characterized by high dimensionality, small sample sizes, and extreme sparsity, rendering models prone to overfitting and making it difficult to capture robust signals (7). Second, and perhaps more critically, traditional ML models (e.g., random forest [RF] and support vector machines) typically treat microbial taxa as independent features. This assumption overlooks the essential phylogenetic dependency among microbes, as taxa are inherently interconnected through an evolutionary tree, sharing genetic backgrounds and functional traits. Neglecting this structural information often results in the underutilization of biological priors embedded in the data, thereby limiting prediction accuracy and generalizability.

To overcome these limitations, researchers have recently attempted to incorporate phylogenetic tree structures into deep learning frameworks. For instance, methods such as PM-CNN (8) and Ph-CNN (9) have utilized convolutional neural networks (CNNs) to extract hierarchical features, whereas DeepPhylo (10) generates microbial vector representations by performing dimensionality reduction on phylogenetic distance matrices. However, a critical limitation persists across these approaches: they predominantly rely on projecting phylogenetic information into Euclidean spaces or utilizing pairwise distances, often neglecting the intrinsic topological connectivity and hierarchical dependencies of the evolutionary tree. Although recent methods, such as Phylo-Spec (11), have made significant progress by utilizing phylogenetic branch lengths within a bottom-up convolution framework, they remain limited by deterministic aggregation strategies. Specifically, Phylo-Spec relies on fixed decay functions (e.g., linear attenuation based on 1 − distance), imposing a “biological hard-coding” that overlooks the task-specific importance of lineages. Consequently, signals from deeply nested taxa could be susceptible to dilution as they traverse hierarchical levels, potentially limiting the preservation of long-range dependencies.

To address these limitations, we propose PhyloGCNE, which reconceptualizes the phylogenetic tree as a graph to enable flexible phylogenetic feature learning. Diverging from the fixed scalar aggregation of previous approaches, our model leverages adaptive information passing facilitated by Graph blocks. Intuitively, each evolutionary branch influences how strongly microbial signals should be shared between connected taxa. This allows the model to preserve the biological structure of the phylogeny while learning which relationships are most informative for the phenotype of interest.

We evaluated the performance of PhyloGCNE on a comprehensive benchmark comprising both a simulated data set and real-world tasks (covering IBD, CRC, T2D, oral squamous cell carcinoma, gastric cancer, dietary fiber intervention, and multiclass predictions). Experimental results demonstrate that PhyloGCNE achieves classification accuracy and robustness superior to existing methods (including Phylo-Spec, DeepPhylo, and RF) across both 16S rRNA and WGS sequencing data. Furthermore, ablation studies confirm that integrating the complete phylogenetic tree structure is critical for enhancing model performance in complex scenarios. PhyloGCNE provides a powerful new tool for leveraging evolutionary information to dissect complex links connecting microbial communities to diverse host physiological states.

## RESULTS

### Overview of the PhyloGCNE framework

**Fig 1:**
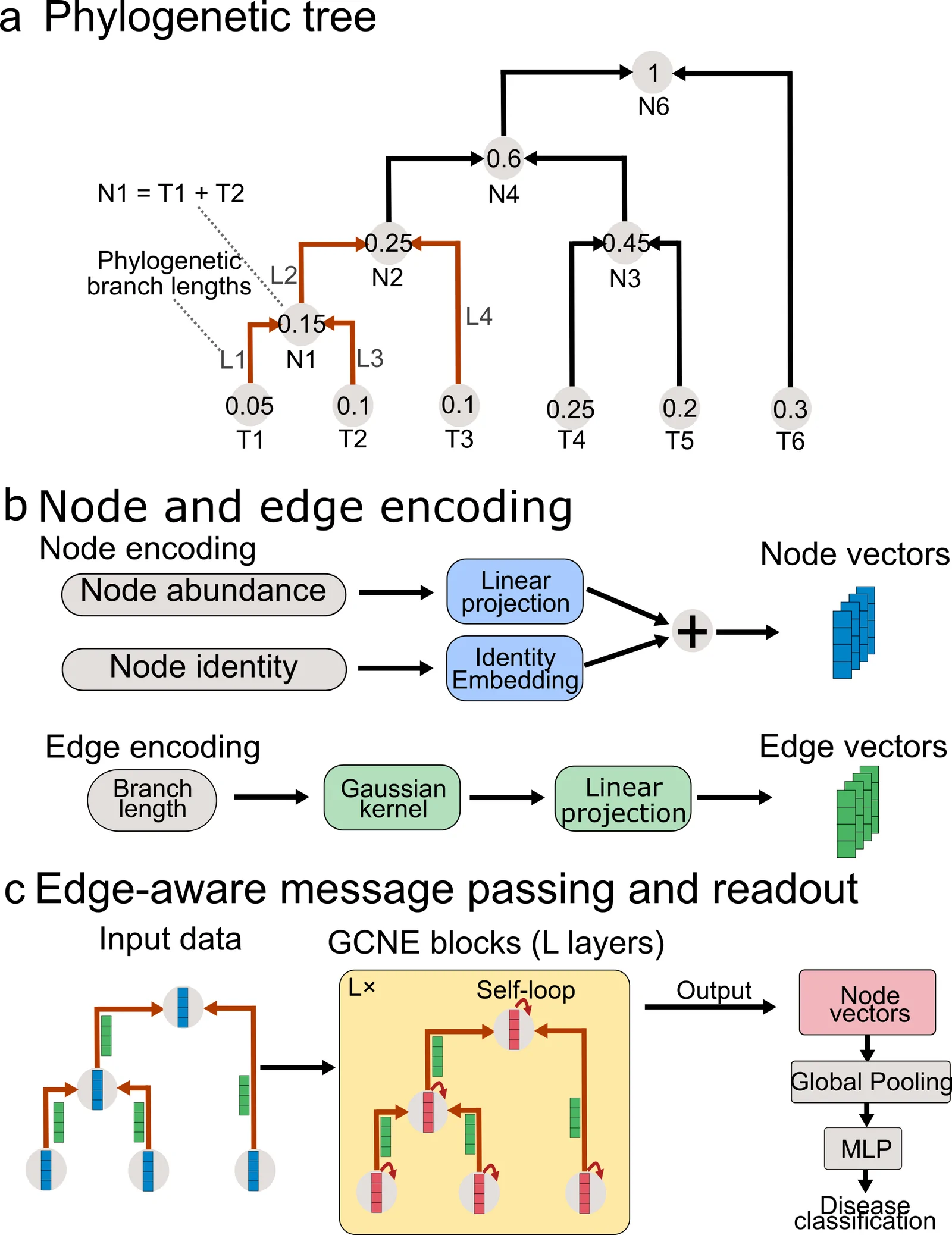
Overall framework of PhyloGCNE. (**a**) Illustration of a microbiome sample incorporating the phylogenetic tree structure. Observed taxa are mapped to the tip nodes, and their values correspond to the relative abundances of each taxon. Parent (internal) node values are defined as the sum of the abundances of their respective child nodes, where T denotes tip nodes, and N denotes internal nodes. (**b**) Node and edge encoding. For node encoding, node abundance and node identity are processed in parallel through a linear projection and an identity embedding, respectively, and the resulting representations are summed to generate node vectors. For edge encoding, each phylogenetic branch length is sequentially transformed by a fixed Gaussian kernel and then by a shared learnable linear projection to generate edge vectors. (**c**) Edge-aware message passing and readout. The encoded input graph is passed through L stacked GCNE blocks, where GCNE denotes graph convolutional network with edge features. Each block updates node representations by aggregating neighboring node information together with edge-vector information, with self-loops included as shown. The resulting node vectors are aggregated by global pooling into a graph-level representation, which is then fed into an MLP for disease classification.

In this study, we introduced PhyloGCNE, a phylogeny-driven deep learning model that integrates microbial evolutionary history, taxonomic composition, and abundance profiles for disease classification. The overall workflow of PhyloGCNE is illustrated in Fig. 1, consisting of three key components: phylogenetic feature representation, graph construction, and deep learning-based classification. First, to capture hierarchical signals within the microbiome, we utilized a phylogeny-based representation strategy (Fig. 1a). Unlike traditional methods that only consider observed taxa (leaf nodes), PhyloGCNE explicitly models ancestral states. Specifically, observed taxa are mapped to the leaf nodes, while internal (parent) nodes are assigned values representing the aggregated abundance of their descendant leaves. This approach allows the model to leverage phylogenetic information at multiple hierarchical levels. Next, node and edge features are encoded separately before graph-based learning (Fig. 1b). Node encoding proceeds through two parallel pathways: node abundance is transformed by a linear projection, whereas node identity is mapped through an identity embedding. The two encoded representations are then summed to generate node vectors. For edge encoding, each phylogenetic branch length is first transformed by a Gaussian kernel, and the resulting value is subsequently passed through a learnable linear projection to generate edge vectors. This design retains an explicit transformation of phylogenetic distance while allowing edge attributes to be adapted during model training. Finally, the disease classification is performed by a graph neural network (GNN) architecture (Fig. 1c). The encoded node and edge representations are fed into a series of stacked GCNE blocks, where GCNE denotes graph convolutional network with edge features. These blocks iteratively update node representations by aggregating information from neighboring nodes together with the corresponding edge features over the phylogenetic graph. After passing through the stacked GCNE blocks, global mean pooling aggregates all node vectors into a graph-level representation, which is then passed to a multilayer perceptron (MLP) for host phenotype classification.

### Validation of the PhyloGCNE model using *in silico* synthetic microbiomes

**Fig 2:**
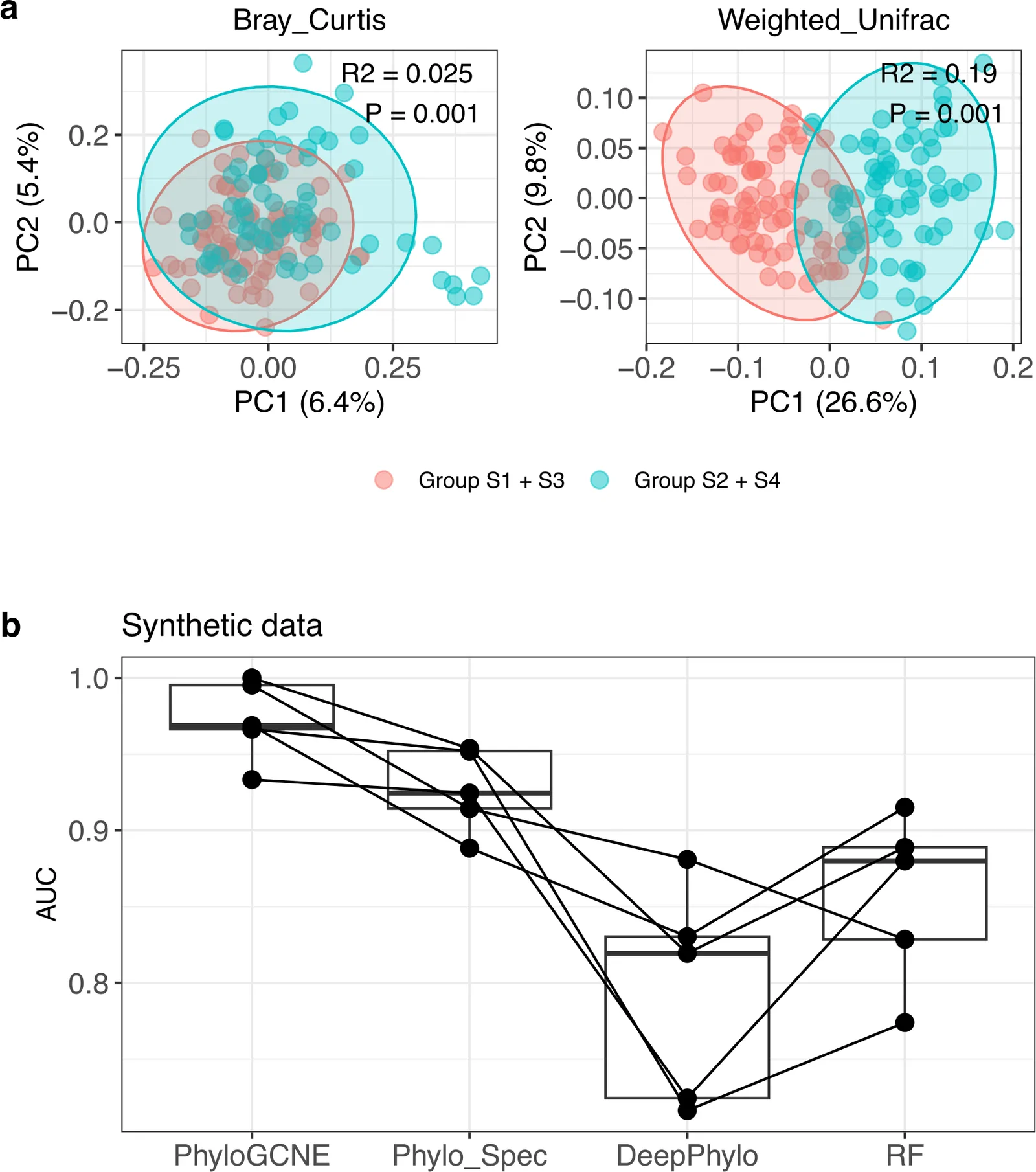
Validation of PhyloGCNE using a synthetic data set with decoupled taxonomic and phylogenetic signals. (**a**) PCoA ordination comparing taxonomy-based (Bray-Curtis) and phylogeny-based (Weighted-UniFrac) clustering. The data set comprises two groups of samples: group 1 (subsets S1 + S3) and group 2 (subsets S2 + S4). S1 and S2 are two subsets with distinct microbial profiles. S3 and S4 are derived from S1 and S2. Specifically, S3 shares a minor fraction of taxa with S2; however, its dominant community structure remains phylogenetically aligned with S1, as reflected by shared evolutionary branches. In contrast, S4 shares only a minor fraction of taxa with S1, while the majority of its composition aligns with S2. Consequently, the abundance-based Bray-Curtis metric shows limited capability to distinguish the groups (*R*^2^ = 0.025), while the phylogeny-aware Weighted-UniFrac metric provides superior group differentiation (*R*^2^ = 0.19). (**b**) Comparison of classification AUCs across different models on the synthetic data set.

To demonstrate the improvement of incorporating phylogenetic information, we utilized the synthetic data set from Zhang et al. (11), which consists of two contrasting groups [group 1 (S1 + S3) and group 2 (S2 + S4)] designed to simulate scenarios where the inter-group variation is fundamentally driven by phylogenetic relationships rather than by distinct taxonomic abundance profiles. We first performed beta-diversity analysis on the synthetic data set using both taxonomy-based (Bray-Curtis) and phylogeny-based (Weighted-UniFrac) metrics. The contrast in clustering patterns was striking: while Bray-Curtis-based PCoA analysis showed substantial overlap between groups (*R*^2^ = 0.025, *P* = 0.001), Weighted-UniFrac revealed a distinct separation with a significantly higher effect size (*R*^2^ = 0.19, *P* = 0.001; Fig. 2a), indicating that variation between groups is fundamentally driven by phylogenetic relationships rather than by taxonomic abundance changes. Consistent with these findings, the classification benchmarks demonstrated the superiority of phylogeny-aware architectures (Fig. 2b). PhyloGCNE achieved the highest performance with a median AUC of 0.97, effectively leveraging the structural signals identified in phylogeny. Notably, PhyloGCNE outperformed Phylo-Spec and significantly surpassed the traditional RF baseline. Interestingly, although DeepPhylo incorporates phylogenetic information, it exhibited high variance and lower median performance compared to RF in this specific synthetic setting, highlighting the robustness of our graph-based modeling strategy.

### PhyloGCNE demonstrates robust generalization across diverse real cohorts

**Fig 3:**
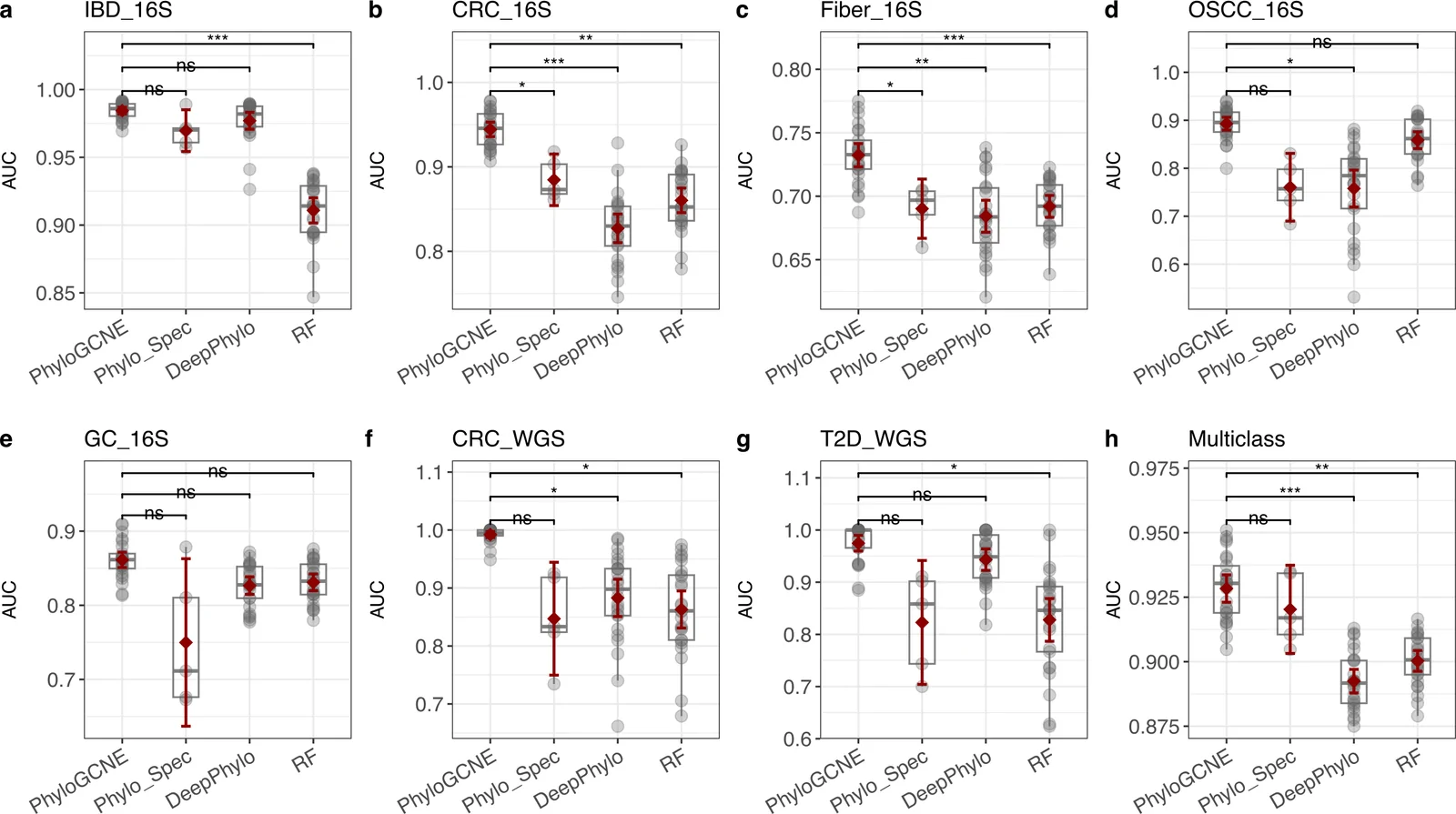
Generalization performance across diverse host phenotype benchmarks. (**a–h**) Comparison of classification AUCs across different diseases and sequencing technologies: (**a**) IBD (16S), (**b**) CRC (16S), (**c**) dietary fiber intervention (16S), (**d**) OSCC (16S), (**e**) GC (16S), (**f**) CRC (WGS), and (**g**) T2D (WGS). (**h**) Classification performance on a multiclass task. Gray dots denote fold-level AUCs from a 5 × 5-fold cross-validation (single stratified fivefold CV for Phylo-Spec), and boxplots summarize the median and interquartile range across folds. Red diamonds indicate the mean AUC across all folds, and red vertical lines indicate 95% *CIs* computed from the fold-level AUC values. Significance of pairwise comparisons between PhyloGCNE and each baseline was assessed using the corrected k-fold cross-validated t-test: ***P < 0.001, *P < 0.01, *P < 0.05, ns: not significant.

Next, we applied PhyloGCNE to real sequencing data sets to benchmark its performance against three baseline methods (Phylo-Spec, DeepPhylo, and RF) across diverse phenotypes and sequencing strategies (16S rRNA and WGS). We first evaluated the model on seven binary benchmark tasks evaluated using matched five repeats of fivefold cross-validation (Fig. 3a through g): IBD (16S), CRC (16S), dietary fiber intervention (16S), oral squamous cell carcinoma (OSCC, 16S), gastric cancer (GC, 16S), CRC (WGS), and T2D (WGS). With the exception of the dietary fiber intervention, OSCC, and GC cohorts, all the benchmark data sets were obtained from the previous work by Zhang et al. (11). Across all these data sets, PhyloGCNE achieved the highest predictive accuracy with mean AUCs ranging from 0.73 to 0.99, including robust performance on the two non-gut cohorts (OSCC: AUC = 0.89 ± 0.03; GC: AUC = 0.86 ± 0.03; Fig. 3d and e), suggesting that the phylogeny-aware graph learning framework may generalize beyond the gut to additional mucosal microbiome settings. Notably, our model significantly outperformed the RF classifier with more stable performance compared to other deep learning methods. In contrast, DeepPhylo showed substantial variability, particularly in the CRC 16S data set (Fig. 3b), where its median AUC dropped below that of the RF, highlighting the advantage of our graph-based aggregation strategy. Next, we extended our evaluation to a complex multiclass data set (16S) also used in the previous work by Zhang et al. (11), where samples were categorized into distinct diagnostic groups (Control, CRC, IBD, IBS, and ASD) (Fig. 3h). In this multiclass classification task, PhyloGCNE achieved a median macro-averaged one-vs-rest AUC of approximately 0.92 and a Macro-F1 of 0.66 (Fig. S1), comparable to Phylo-Spec (AUC ≈ 0.94; Macro-F1 = 0.66) and substantially outperforming DeepPhylo (Macro-F1 = 0.47). Per-class confusion matrices (Fig. S1) further reveal that while Phylo-Spec and RF achieve higher recall for individual disease classes, this comes at the cost of severely reduced Control recall (Phylo-Spec: 0.52; RF: 0.29). PhyloGCNE maintains the most balanced per-class recall across all five categories (Control: 0.72; IBD: 0.80; ASD: 0.72; CRC: 0.56; IBS: 0.71). Collectively, these results indicate that PhyloGCNE captures robust, status-associated phylogenetic signals that are transferable across diverse biological contexts and sequencing technologies. The corrected resampled *t*-tests (see Materials and Methods) confirmed that PhyloGCNE achieved statistically significant improvements over RF and DeepPhylo across the majority of data sets (Fig. 3). Although PhyloGCNE also showed consistently higher mean AUCs than Phylo-Spec across all cohorts, most of these comparisons did not reach statistical significance. To further test robustness against inter-study batch effects, we performed a Leave-One-Dataset-Out (LODO) evaluation on the CRC 16S cohorts. In each iteration, the model was trained on N − 1 cohorts and evaluated on the remaining held-out cohort. As shown in Fig. S2, PhyloGCNE maintained high AUCs across most held-out cohorts and showed lower inter-cohort performance variation than the other methods. In contrast, RF exhibited substantial instability under cross-study transfer, with its AUC dropping to approximately 0.46 on PRJNA911189. These findings suggest that the phylogeny-aware feature aggregation strategy of PhyloGCNE improves robustness and generalizability across independent study cohorts.

### Impact of phylogenetic information on diverse clinical data sets

**Fig 4:**
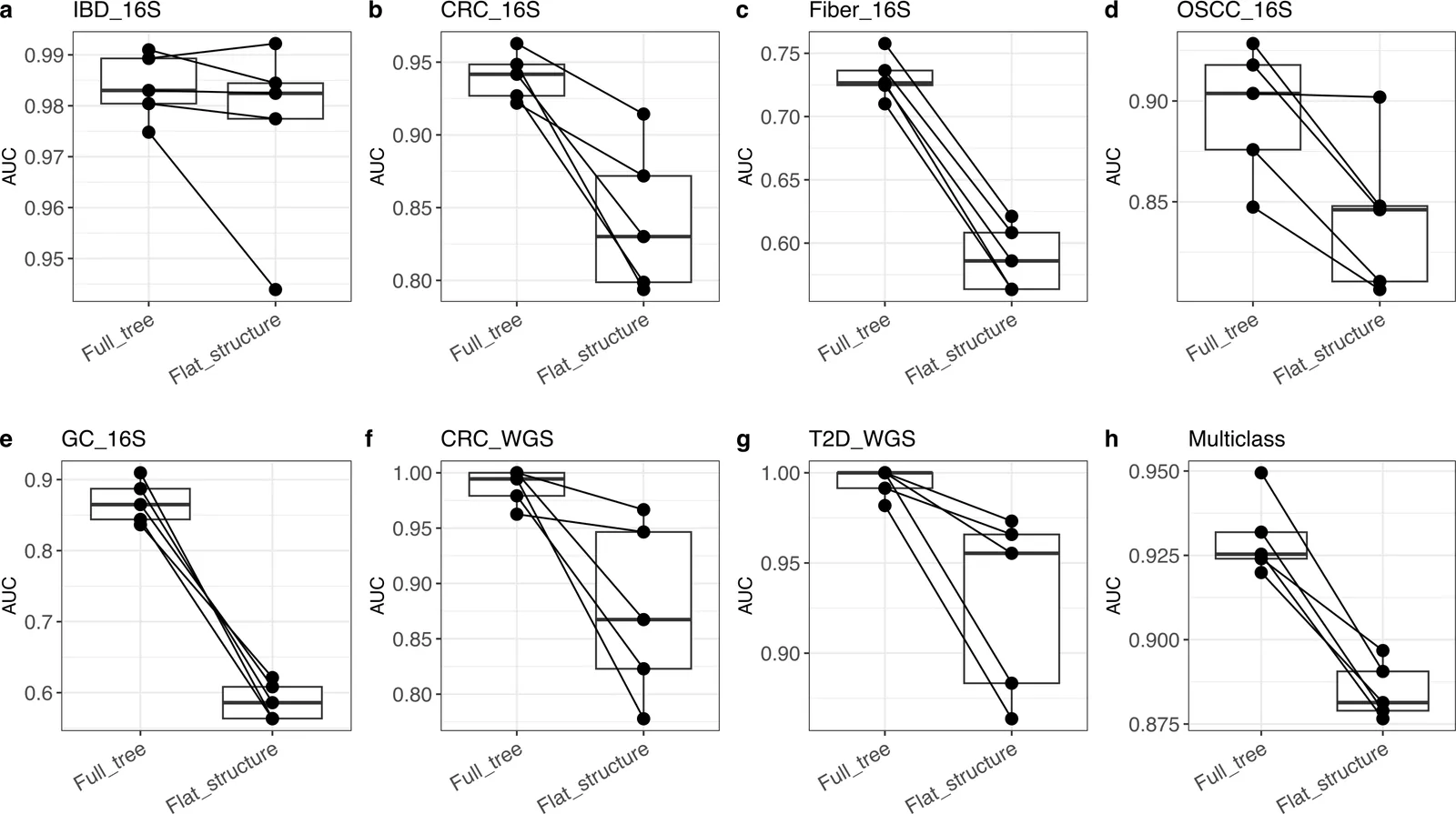
Impact of phylogenetic information on the classification performance of PhyloGCNE. We compared the full tree graph with a flat structure variant to evaluate the contribution of evolutionary structures. The flat structure ablation uses a star topology (all leaf taxa connected directly to the root, internal nodes removed), retains leaf abundances unchanged, sets all branch lengths uniformly to 1, and keeps the GNN architecture and training protocol identical to the Full Tree condition. A schematic is provided in Fig. S3. (**a–h**) Comparison of classification AUCs across different diseases and sequencing platforms: (**a**) IBD (16S), (**b**) CRC (16S), (**c**) dietary fiber intervention (16S), (**d**) OSCC (16S), (**e**) GC (16S), (**f**) CRC (WGS), and (**g**) T2D (WGS). (**h**) Classification performance on a multiclass data set. Black dots denote fold-level AUCs from matched stratified five-fold cross-validation, and boxplots summarize the median and interquartile range across folds. Lines connecting boxplots represent paired observations across matched fivefold cross-validation splits (seed = 42). Because internal nodes are removed in the flat-structure variant, the total number of node embeddings and therefore the exact parameter count differ from the full-tree model; all other model hyperparameters and training settings were kept identical.

We next investigated how much phylogenetic information contributed to model performance (Fig. 4). Although the influence of phylogeny was preliminarily explored in the simulation data set (Fig. 2), it is critical to validate these findings in real-world settings, where microbiome data are sparse, noisy, and ecologically heterogeneous. Figure 4 compares two distinct graph construction strategies: the complete phylogenetic tree versus a flat graph structure consisting solely of leaf nodes directly connected to the root. Across most cohorts, the flat structure caused a substantial decline in classification performance. The most pronounced degradation was observed in GC (16S) (Fig. 4e), where the median AUC dropped by approximately 0.26, suggesting that gastric microbiome disease signals are highly dependent on quantitative evolutionary structure. Marked declines were also observed in CRC (16S and WGS), T2D (WGS), dietary fiber intervention (16S), and the multiclass task. Performance degradation under the flat structure was also evident in OSCC (16S) (Fig. 4d), though to a lesser extent than GC (16S). An exception to this trend is observed in the IBD (16S) data set (Fig. 4a), where the performance gap between the full tree and the flat structure is negligible, suggesting that for the IBD cohort, the discriminative signals may be predominantly driven by abundance variations of individual taxa (leaf nodes), rather than by hierarchical phylogeny. Despite this dataset-specific variation, the overall degradation in performance observed with phylogeny ablation highlights the critical contribution of hierarchical evolutionary information to the model classification.

### Identification and validation of PhyloGCNE-derived microbial features

Having established the critical role of phylogenetic structures in enhancing classification performance, we next sought to decipher the biological signals driving PhyloGCNE’s predictions. A key challenge in deep learning for microbiomes is interpretability. To address this, we developed the Phylogenetic Saliency Propagation (PSP, see Materials and Methods) framework, which integrates our novel Bio-Constrained Adaptive SmoothGrad (BAS-Grad) with top-down evolutionary propagation to quantify the contribution of specific leaf taxa.

**Fig 5:**
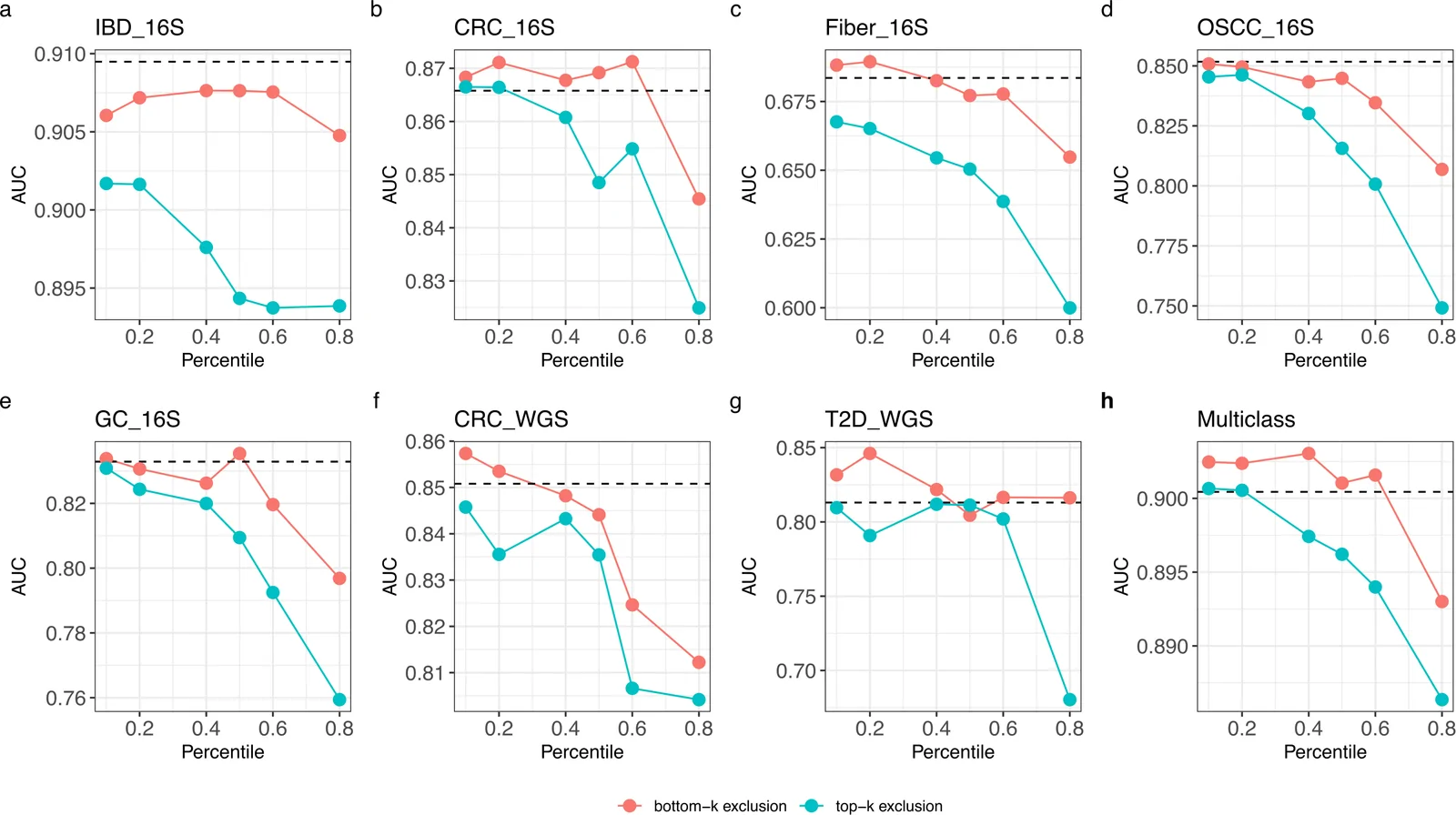
Validation of feature importance identified by PhyloGCNE on real-world data sets. We removed a specific percentage of features (x-axis) ranked as either the most important (top-k exclusion, teal) or the least important (bottom-k exclusion, pink) by PhyloGCNE. The remaining features were then used to train and test an RF classifier. The dashed horizontal line represents the baseline AUC using the complete feature set. The panels show validation results across different data sets: (**a**) IBD (16S), (**b**) CRC (16S), (**c**) dietary fiber intervention (16S), (**d**) OSCC (16S), (**e**) GC (16S), (**f**) CRC (WGS), (**g**) T2D (WGS), and (**h**) a multiclass data set.

To rigorously validate whether the taxa identified by PSP are indeed biologically significant, we conducted a progressive feature elimination analysis (Fig. 5). We stratified microbial features based on their PSP-derived importance scores and established two experimental settings: “top-k exclusion” (iteratively removing the most important features) and “bottom-k exclusion” (removing the least important features). Crucially, to ensure an unbiased and model-agnostic evaluation, the discriminative power of the remaining feature subsets was tested using an independent RF classifier, rather than the PhyloGCNE model itself.

The results demonstrated a divergence in performance degradation between the two groups across all data sets. As illustrated in Fig. 5, removing the top-ranked features (teal curves, “top-k exclusion”) led to a precipitous decline in classification AUC relative to the baseline (all features, dashed line). For instance, in the IBD (16S) (Fig. 5a), fiber intervention (16S) (Fig. 5c), and CRC (WGS) (Fig. 5f) cohorts, the AUC dropped after excluding only the top 20% of identified features, confirming that these taxa contain the majority of the host phenotype-associated information. Notably, the two non-gut cohorts, OSCC (16S) (Fig. 5d) and GC (16S) (Fig. 5e), exhibited similarly pronounced top-k sensitivity, with teal curves declining steeply upon removal of top-ranked features, suggesting that PSP successfully captures disease-relevant biomarkers even in non-gut microbiome contexts.

In contrast, removing the low-ranked features (pink curves, “bottom-k exclusion”) had a negligible impact on model performance. In data sets such as T2D (WGS) (Fig. 5g) and the multiclass task (Fig. 5h), the AUC remained stable or even slightly improved initially, suggesting that the PSP framework correctly identified and assigned low scores to irrelevant or noisy taxa. The performance only began to degrade when a very large proportion (>60%–80%) of the data was removed. This validation confirms that PhyloGCNE, equipped with the PSP interpretation strategy, successfully isolates robust, biologically meaningful biomarkers from high-dimensional microbiome data.

**Fig 6:**
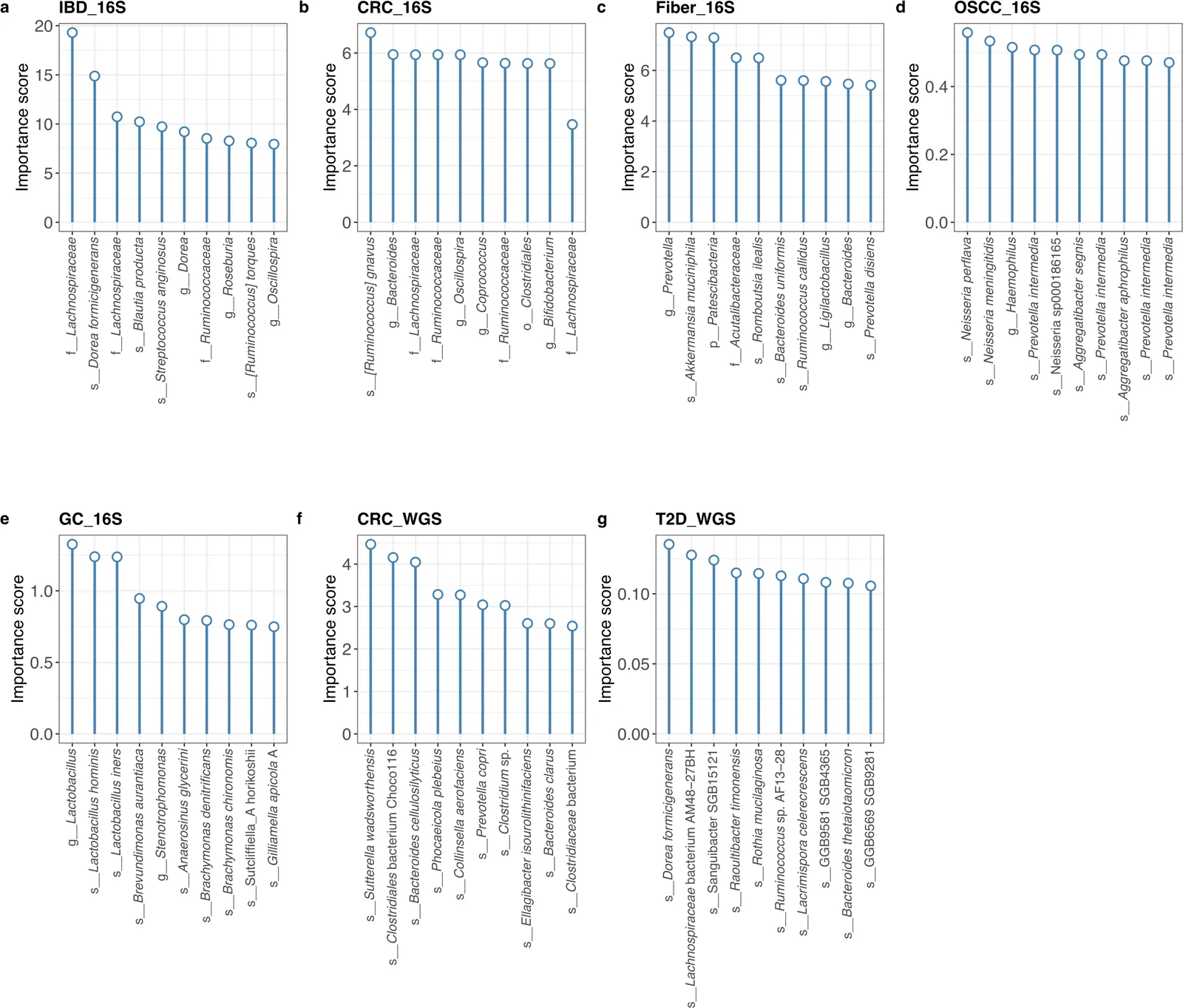
Significant biomarkers identified by PhyloGCNE. The bar plots display the top leaf nodes of microbial taxa ranked by their importance scores (derived from the PSP framework) for each data set. (**a**) IBD (16S), (**b**) CRC (16S), (**c**) dietary fiber intervention (16S), (**d**) OSCC (16S), (**e**) GC (16S), (**f**) CRC (WGS), and (**g**) T2D (WGS).

To elucidate PhyloGCNE’s biological interpretability, we analyzed the top-ranked features across data sets (Fig. 6). In IBD (Fig. 6a), the model prioritized f__*Lachnospiraceae* and g__*Roseburia*, which include many butyrate-producing taxa and are often depleted in IBD (1, 12), alongside the mucin-degrader s__*Eubacterium_torques* (13). For CRC (Fig. 6b), it identified the pro-inflammatory s__*Ruminococcus_gnavus* (14), as well as beneficial genera (g__*Oscillospira* and g__*Coprococcus*) known to be depleted in disease (15, 16). Notably, distinct features belonging to f__*Lachnospiraceae* were consistently prioritized in both IBD and CRC cohorts, highlighting the model’s ability to capture the depletion of this core butyrate-producing family as a shared signature of intestinal inflammation. The WGS results (Fig. 6f) offered superior resolution, pinpointing the IgA-degrading s__*Sutterella wadsworthensis* (17) and the pro-inflammatory s__*Collinsella aerofaciens* (18), a pathobiont associated with disease susceptibility. Regarding dietary fiber intervention (Fig. 6c), g__*Prevotella* emerged as a primary responder (4), along with a beneficial set of microbes including s__*Akkermansia muciniphila*, s__*Bacteroides_uniformis*, and g__*Ligilactobacillus* (19–21). Finally, in T2D (Fig. 6g), the model highlighted the T2D-enriched s__*Dorea formicigenerans* (22) and the oral commensal s__*Rothia mucilaginosa* reflecting disease-associated oral-gut transmission (23, 24). For OSCC (Fig. 6d), PhyloGCNE consistently prioritized oral-associated pathobionts, with s*__Prevotella intermedia* appearing among the highest-ranked features, consistent with its established role as an enriched species in the tumor-associated oral microbiome of OSCC patients (25). For GC (Fig. 6e), g*__Lactobacillus* species (s*__Lactobacillus hominis*, s*__Lactobacillus iners*) dominated the top-ranked features, consistent with previous reports of altered *Lactobacillus* abundance as a discriminative signature in gastric cancer microbiome studies (26, 27). The concordance of PhyloGCNE-identified biomarkers with established literature across both non-gut cohorts further validates the model’s capacity to capture disease-associated microbiome shifts beyond the gut environment. To provide complementary community-level context alongside the taxon-level analysis, we performed co-occurrence network analysis on the T2D cohort using ggClusterNet 2. Compared to healthy controls, the T2D network exhibited reduced connectivity, with fewer active nodes (30 vs. 42), fewer edges (36 vs. 61), a more than 50% reduction in average clustering coefficient (0.206 vs. 0.448), and a complete absence of negative correlation edges (Fig. S4 Table S3), indicating an overall reduction in microbial co-occurrence associations under T2D conditions.

Collectively, these findings confirm that PhyloGCNE does not merely rely on “black-box” patterns but effectively leverages the phylogenetic structure to capture robust, biologically interpretable mechanisms driving host phenotypes.

## DISCUSSION

In this study, we presented PhyloGCNE, a phylogeny-driven graph convolutional network designed to decipher complex links connecting microbial communities to diverse host phenotypes. By reconceptualizing the phylogenetic tree as a graph structure with learnable projections of Gaussian-kernelized edge attributes, PhyloGCNE effectively addresses the challenges of high dimensionality and sparsity inherent in microbiome data. Our comprehensive benchmarking across diverse cohorts (IBD, CRC, T2D, OSCC, GC, and dietary fiber intervention) and sequencing platforms (16S rRNA and WGS) demonstrates that PhyloGCNE not only achieves state-of-the-art classification accuracy but also provides robust and biologically meaningful interpretability.

A key finding of our work is that explicit modeling of phylogenetic relationships significantly enhances predictive performance. While traditional machine learning models (e.g., RF) and some deep learning approaches, such as DeepMicro (28) and MDL4Microbiome (29)**,** treat microbial taxa as independent features, our ablation study (Fig. 4) revealed that removing phylogenetic structure (the flat structure) leads to a marked decline in performance. This suggests that the host phenotype-associated signals are not confined to individual species but are distributed across broader taxonomic lineages, consistent with the ecological theory of phylogenetic conservatism of functional traits (30), where closely related taxa often share similar pathogenic potential or metabolic functions driven by environmental filtering (31, 32). By enabling information propagation through ancestral nodes, PhyloGCNE effectively captures these clade-level signals. By identifying functional associations between microbes rather than treating them as isolated entities, the model provides a more accurate representation of the host phenotype than approaches based on single species.

One of the most persistent criticisms of deep learning in medicine is the “black box” nature of the models (33). It is widely observed that high accuracy is often achieved at the expense of interpretability, creating a barrier to clinical trust and implementation (34). To overcome this, we developed the PSP framework, a novel interpretation strategy that respects the evolutionary dependencies of the input data. The validation results in Fig. 5 provide compelling evidence for the reliability of this approach. The perturbation analysis demonstrated that removing the features identified as “important” by PSP caused a precipitous drop in classification AUC, whereas removing low-ranked features had minimal impact. This distinct separation confirms that PhyloGCNE is not merely learning statistical artifacts or noise, but is successfully locking onto the true microbial drivers of the host phenotype. Notably, *Fusobacterium nucleatum*, a well-established CRC-associated taxon, did not emerge as a top-ranked biomarker in our analysis. This might reflect limited 16S species-level resolution within *Fusobacterium* (35) and subspecies-level dilution in WGS, as CRC enrichment is driven specifically by *F. nucleatum subsp. animalis* clade 2 (36). This is consistent with its non-significant species-level differential abundance in the WGS cohort (LinDA *P*-adj = 0.057; MaAsLin2 *P*-adj = 0.113).

Despite its promising performance, our study has specific limitations rooted in its exclusive reliance on phylogenetic topology. First, our model operates on the premise of phylogenetic conservatism, assuming that functional traits are vertically inherited along the evolutionary tree. This structural prior may limit the detection of phenotypes driven by horizontal gene transfer (HGT), such as plasmid-mediated antibiotic resistance, where functional capacity is decoupled from species-level evolutionary history. Second, the graph connectivity in PhyloGCNE is strictly defined by ancestral relationships, which means the model explicitly captures signal propagation between evolutionary neighbors but may overlook ecological interactions between phylogenetically distant taxa. Complex microbiome dynamics often involve metabolic cross-feeding or competitive exclusion across disparate lineages (e.g., syntrophy between different phyla), which are not represented in the current tree-based graph structure. Future iterations could address this by integrating ecological co-occurrence networks or metabolic pathway data to construct dual-channel graphs. Third, the current study does not assess the calibration of predicted probabilities. For clinical scenarios where predicted scores inform risk stratification or treatment decisions, well-calibrated outputs are essential. Future work will incorporate calibration evaluation and post hoc calibration techniques to support clinical translation. Additionally, future work will extend validation to underexplored body sites, including the bladder and skin microbiome, building upon recent advances in catheterized-urine sampling and contamination control (37).

In conclusion, PhyloGCNE represents a significant step forward in phylogeny-aware deep learning. By seamlessly integrating evolutionary history with deep learning, it offers a powerful framework for accurate host phenotype classification and the discovery of microbial biomarkers, potentially accelerating the translation of microbiome research into clinical and nutritional practice. In particular, the model’s demonstrated utility on a healthy-population dietary fiber intervention cohort highlights its applicability beyond disease diagnosis. As articulated by this perspective (5), the microbiome plays critical roles not only in human disease but also in nutrition, food safety, and environmental health, and understanding these roles requires scalable computational tools that can operate across diverse study designs and body sites. PhyloGCNE’s design, accepting any rooted phylogenetic tree as input regardless of habitat origin or sequencing platform, positions it as a candidate analytical module within such integrated frameworks. Moreover, the PSP interpretation layer can identify conserved or site-specific microbial signatures across different host environments, potentially facilitating cross-domain biomarker translation. Looking forward, coupling PhyloGCNE with multi-omics data streams (e.g., metatranscriptomics or metabolomics) as advocated by the integrated meta-omics paradigm could further enhance both predictive accuracy and mechanistic understanding of host–microbiome interactions across the One Health continuum.

## MATERIALS AND METHODS

### Phylogenetic graph construction and feature initialization

To effectively incorporate evolutionary information into the learning process, we constructed the graph topology explicitly based on the phylogenetic tree structure. Let *T* = (*V, E*) represent the phylogenetic tree, where *V* denotes the set of all nodes, including leaf taxa and internal ancestral nodes, and *E* represents the branches connecting them.

#### Node abundance initialization

For each sample i, the initial feature vector for the leaf nodes was assigned based on their relative abundances derived from the taxonomy profile table. To prevent information loss from the hierarchical structure, we employed a bottom-up aggregation strategy for internal nodes. Specifically, the abundance of an internal node *V* was calculated as the sum of the abundances of its direct children:


$$x_{v}=\left\{ \begin{array}{ll} A_{v}, & \text{if }v\text{ is a leaf node},\\ \sum_{u\in children(v)}x_{u}, & \text{otherwise}. \end{array}\right.$$

where *A_v_* represents the observed abundance of taxa *v*. This process ensures that higher-level taxonomic nodes represent the aggregated abundance of their evolutionary descendants.

#### Phylogenetic distance-based edge attribute

The edges in our graph correspond to the branches of the phylogenetic tree. To encode evolutionary relatedness between connected nodes, we transformed the normalized phylogenetic distance *d_uv_* between node *u* and *v* into an edge coefficient *c_uv_* using a Gaussian kernel, following the strategy adopted in PM-CNN (8):


$$c_{uv}=exp\left(-2\rho d_{uv}^{2}\right)$$

where ρ is a scaling hyperparameter (set to 2 in our experiments). These edge weights serve as input attributes for the edge-aware message-passing layers.

This transformation is motivated by the concept of phylogenetic covariance, where closely related taxa are expected to share more similar traits because of common evolutionary history (38). Consistent with the covariance-decay principle, we mapped branch lengths into bounded coefficients, so that nearby nodes receive stronger edge attributes and distant nodes receive weaker ones. Thus, *c*_*uv*_ provides a biologically motivated representation of evolutionary relatedness for the edge-aware message-passing layers. The edge coefficient *c*_*uv*_ serves as input to a learnable linear projection, which produces a *d*-dimensional edge embedding that adaptively modulates phylogenetic signals during message passing.

### PhyloGCNE model architecture

PhyloGCNE is a deep learning framework designed to learn representations from the constructed phylogenetic graphs.

#### Learnable node embeddings

To capture intrinsic taxa-specific characteristics distinct from abundance metrics, a learnable embedding was assigned for each node. This feature embedding is subsequently integrated with the abundance feature to yield the input representation $h_{v}^{\left(0\right)}$, computed by:


$$h_{v}^{\left(0\right)}=W_{in}x_{v}+E_{id}[v]$$

where *x_v_* is the abundance, $W_{in}\in R^{1\times d}$ is a linear projection matrix, and $E_{id}\in R^{\left|v\right|\times d}$ is a lookup table for learnable node embeddings of dimension *d*.

#### Edge-aware message passing

Standard GCNs typically operate on node features while utilizing edges only for connectivity (39). To fully leverage the phylogenetic branch lengths, we designed a custom message-passing layer that explicitly incorporates edge attributes into the feature propagation. The propagation rule for node *v* at layer *l* is defined as:


$$h_{v}^{\left(l+1\right)}=\sum_{u\in N\left(v\right)\cup \left\{v\right\}}a_{uv}\cdot \left(W_{node}^{\left(l\right)}h_{u}^{l}+W_{edge}^{\left(l\right)}c_{uv}\right)$$

where *N*_(_*_v_*_)_ denotes the neighbors of node *v*, and *a_uv_* is the normalization constant derived from the degree matrix (symmetric normalization $\frac{1}{\sqrt{deg\left(v\right)\;deg\left(u\right)}}$). *W*_node_ and *N*_(_*_v_*_)_ are learnable weight matrices for node features and edge attributes, respectively. This mechanism ensures that the message passed from a neighbor is modulated by both the neighbor’s state and the evolutionary distance connecting them.

#### Implementation details of graph construction

Self-loops are added to each node with an edge attribute of zero. Because the edge transformation layer contains no bias term, a zero-valued edge attribute produces a zero-edge embedding, ensuring that self-connections contribute no phylogenetic distance signal and are governed exclusively by the node transformation pathway. The phylogenetic tree is converted into a directed graph with edges pointing from each child node to its parent (child→parent). All nodes, including internal (ancestral) nodes, are included for every data set. Leaf node features are set to the observed taxon abundance; internal node features are assigned the sum of all descendant leaf abundances. The initial edge coefficient *c*_*uv*_ is obtained by applying a Gaussian kernel to the branch length and is used as a one-dimensional edge attribute. This value is subsequently projected into a d-dimensional edge embedding by a learnable linear layer within the GCNE block, as described above.

#### Stacked GCNE blocks and classification

The PhyloGCNE architecture is constructed by stacking multiple GCNE blocks, adopting the design principles proposed by Luo et al. (40). Each block sequentially applies the edge-aware message-passing layer followed by normalization (batch normalization or layer normalization), a non-linear activation function (ReLU), and Dropout for regularization. To facilitate gradient propagation across deep layers, we employ a residual skip connection. The computation for the *l*+1-th layer is defined as


$$H^{\left(l+1\right)}=Dropout\left(\sigma \left(Norm\left(Conv\left(H^{\left(l\right)},E\right)\right)\right)\right)+H^{\left(l\right)}$$

Following *l* layers of message passing, a global mean pooling aggregates node representations into a graph-level vector *h_G_*. Finally, a multi-layer perceptron (MLP) classification head projects *h*_*G*_ to the prediction score for the target phenotype.

### Feature importance estimation via PSP

To delineate the specific microbial taxa (leaf nodes) driving the model’s predictions, we developed a two-stage interpretation framework named PSP. PSP should be distinguished from the predictive message passing in PhyloGCNE. In the GCNE block, phylogenetic edges are directed from child to parent, so messages pass from descendant nodes to their immediate ancestors under the PyTorch Geometric source → target convention. This bottom-up aggregation allows internal nodes to summarize signals from descendant subtrees before graph-level readout. By contrast, PSP is a *post hoc* interpretation procedure applied after model training. It propagates saliency from ancestral lineages downward to descendant leaf taxa, converting lineage-level importance into taxon-level biomarker rankings. Thus, bottom-up message passing supports predictive representation learning, whereas top-down PSP supports interpretable attribution. Specifically, PSP employs a dual-stage mechanism that first anchors importance scores in robust, data-driven local sensitivity via Bio-Constrained Adaptive SmoothGrad (BAS-Grad), and subsequently enriches these signals by propagating evolutionary context from ancestral lineages down to individual leaf taxa.

1. BAS-Grad: Standard gradient-based interpretation methods (e.g., Saliency Maps) often suffer from gradient saturation and sensitivity to local noise. While SmoothGrad (41) mitigates this by averaging gradients over noisy inputs, its assumption of uniform Gaussian noise is ill-suited for microbiome data, which is characterized by high sparsity, non-negativity, and heteroscedasticity. To address this, we developed Bio-Constrained Adaptive SmoothGrad (BAS-Grad), which introduces two critical domain-specific regularizations:
  (1) Instead of applying a uniform noise scale, we employ a dynamic perturbation strategy. For a given input batch *X*, a bounded random variable *z* was sampled from a Beta distribution, *z* ~ Beta(α = 2, β = 5), and then mean-centered as *z*′ = *z* − α/(α + β). Because α/(α + β) is the expected value of the Beta distribution, this transformation yields a bounded perturbation with zero expectation. The perturbation applied to taxon *k* was then defined as ε*_k_* = λσ*_k_z*′, where σ*_k_* denotes the standard deviation of taxon *k* across samples and λ = 0.15 controls the overall perturbation scale. This ensures that perturbation magnitudes are proportional to the natural variability of each taxon, preventing the masking of signals from low-abundance species.
  (2) To respect the strict non-negativity of abundance data, we enforce a rectification step: $\tilde{X}=max(X+\varepsilon ,0)$. This constraint ensures that gradient estimation occurs strictly within the biologically valid data manifold.
    For a specific sample *j*, we generated *N* = 50 perturbed copies. The importance score *S_i_* node *i* was calculated by averaging the gradients accumulated over these noisy samples:

$$S_{i,j}^{base}=\frac{1}{N}\sum_{k=1}^{N}\left(\frac{\partial f\left(\tilde{X_{k}}\right)}{\partial \tilde{X_{ki}}}\right)$$

where f(∙) represents the predicted score for the target class. Finally, the importance scores were aggregated globally by averaging the score values for each node across all samples by $S_{i}^{base}=\frac{1}{M}\sum_{j=1}^{M}S_{i,j}^{base}$.

**Table:** 

Algorithm 1. BAS-Grad importance calculation
1: Input: 2: Model f: The trained PhyloGCNE model 3: Input X: A batch of abundance profiles # [Batch_Size, Num_Taxa] 4: N: Number of noisy samples per input (*N* = 50) 5: sigma_spread: Noise scaling factor (sigma_spread = 0.15) 6: alpha = 2, beta = 5: Parameters for Beta distribution 7: Output: 8: S: Importance scores (Saliency Map) for input taxa 9: Procedure: 10: sigma[k] =StdDev(X[:, k]) # Calculate standard deviation sigma for each taxon k 11: S_accumulated = Zeros_Like(X) 12: For k = 1 to N do: 13: noise_raw ~ Beta(alpha, beta) - alpha/(alpha + beta) 14: epsilon = noise_raw * sigma * sigma_spread 15: X_perturbed = Max(X + epsilon, 0) 16: gradient_k = Gradient(f_c(X_perturbed), X_perturbed) 17: S_accumulated = S_accumulated + gradient_k 18: End For 19: S = S_accumulated / N 20: Return S

1. Top-down importance propagation: To quantify the cumulative contribution of each leaf node, we implemented a top-down propagation mechanism. We hypothesize that the importance of a specific microbe is derived not only from its individual signal but also from the evolutionary constraints of its lineage. For any node *u* and its parent *p*, the final importance score $S_{u}^{final}$ is updated recursively:

$$S_{u}^{final}=S_{u}^{base}+\left(S_{p}^{final}\times \emptyset \left(d_{u,p}\right)\right)$$

where *d* represents the patristic distance (branch length) between node *u* and its parent *p*. The term ∅(∙) is a Gaussian decay kernel defined as $\emptyset \left(d\right)=exp\left(-2\rho d^{2}\right)$ with a scaling parameter ρ=2. The same ρ from edge construction is used here. This formulation ensures that importance from ancestral nodes “propagates” down to their descendants, ensuring that the identified biomarkers exhibit phylogenetic coherence, prioritizing taxa that are supported by the evolutionary context of their lineage rather than relying solely on isolated artifacts.

1. Leaf node ranking: After propagation, the final importance scores of the leaf nodes integrate both local feature sensitivity and hierarchical phylogenetic signals. We extracted and ranked these leaf-node scores to identify the most significant microbial biomarkers associated with the prediction.

### Data sets and benchmark

The proposed PhyloGCNE model was implemented using the PyTorch framework in Python. For comparative analysis, benchmark deep learning methods, including Phylo-Spec (11) and DeepPhylo (10), were executed using their officially provided source code. The RF algorithm was implemented using the scikit-learn library (42). To comprehensively evaluate the effectiveness of PhyloGCNE, we utilized one simulated data set and eight real-world microbiome cohorts: IBD (16S), CRC (16S), dietary fiber intervention (16S), oral squamous cell carcinoma (OSCC, 16S), gastric cancer (GC, 16S), CRC (WGS), T2D (WGS), and a multiclass data set. With the exception of the dietary fiber intervention, OSCC, and GC cohorts, all data sets were directly obtained from the previous work by Zhang et al. (11). The complete data set characteristics, including accession numbers, sample sizes for each cohort, phenotype-stratified total sample sizes, and the number of microbial features, are summarized in Table S1. Raw sequencing data are publicly accessible through the respective NCBI SRA repositories, and the associated metadata used in this study have been consolidated and made available in our GitHub repository. For the dietary fiber intervention (16S), OSCC (16S), and GC (16S) data sets, the raw sequences were processed using the DADA2 pipeline to infer amplicon sequence variants (ASVs) (43). Subsequently, given that the included studies targeted different hypervariable regions of the 16S rRNA gene, closed-reference clustering was performed at 99% sequence identity against the Greengenes2 (44) database using the QIIME 2 vsearch plugin (45). The metadata associated with the OSCC (16S) and GC (16S) data sets were obtained from the original studies conducted by Han et al. (46) and Li et al. (47), respectively.

The classification performance of all models was assessed using the area under the receiver operating characteristic curve (AUC) as the primary metric. To improve the transparency of performance reporting, we summarized fold-level variability across five repeats of fivefold cross-validation protocol; the only exception was Phylo-Spec (deep learning model), which was evaluated under a single stratified fivefold CV due to the substantial computational cost of its CPU-only implementation. Unless otherwise stated, values are reported as mean ± standard deviation (SD) across folds. For Fig. 3, 95% confidence intervals (CIs) were computed from 25 fold-level AUC values (or five values for Phylo-Spec). Pairwise comparisons between PhyloGCNE and each baseline were performed using the corrected k-fold cross-validated t-test proposed by Nadeau and Bengio (48, 49), implemented via the kfold_ttest function in the correctR R package (50), on matched fold-level AUC values. For the multiclass classification task, model performance was evaluated using macro-averaged one-vs-rest AUC, Macro-F1, Macro-Precision, and per-class recall. As Phylo-Spec is included in the multiclass comparison, all models were evaluated under the same single stratified fivefold CV. Per-class recall was derived from the row-normalized confusion matrices, where each row represents the distribution of predicted labels for samples belonging to the corresponding true class.

### Reference phylogeny selection and pruning

A single phylogenetic tree was constructed for each data set and shared across all phylogeny-aware methods (PhyloGCNE, Phylo-Spec, and DeepPhylo), while RF, as a phylogeny-agnostic method, did not utilize tree information. All trees were obtained from curated reference databases. For the 16S rRNA benchmark data sets (IBD, CRC, and multiclass) from Zhang et al. (11), the reference phylogenetic tree was directly obtained from the original publication, which used Greengenes reference tree (v13.8). For the WGS metagenomic benchmark data sets (CRC and T2D), also obtained from Zhang et al. (11), the corresponding reference phylogenetic tree provided by MetaPhlAn4 was used. For the dietary fiber intervention (16S), OSCC (16S), and GC (16S) data sets, the reference phylogenetic tree was obtained from the Greengenes2 database (44). In all cases, each reference tree was pruned to retain only the taxa present in the respective data set using the phyloseq R package (51), yielding a phylogenetically consistent subtree as a direct subset of the reference topology. Because branch lengths varied substantially in numerical range across the reference trees, max root-to-tip normalization was applied to all trees prior to Gaussian transformation, with every branch length divided by the maximum root-to-tip distance of the respective tree to scale all values to (0, 1]. This preserves the relative proportions among branch lengths within each tree while standardizing the input scale across data sets.

### Model training and evaluation protocol

All real-world data sets were evaluated using a 5 × 5-fold cross-validation procedure resulting in 80% training data and 20% test data in each fold. Hyperparameters were selected using the *in silico* synthetic data set as a development benchmark and then fixed before evaluation on all real-world data sets, thereby avoiding dataset-specific tuning on real-world test folds. For each real-world data set, model evaluation was performed using matched stratified fivefold cross-validation. Within each training fold, 10% of the training samples were further stratified as an internal validation set for early stopping. PhyloGCNE was trained for up to 100 epochs, and training was stopped if the validation loss did not improve for five consecutive epochs. The held-out test fold was used only for final performance evaluation and was never used for model selection, hyperparameter tuning, or early stopping. The complete grid-search space and final hyperparameter configurations are provided in Table S2. To complement the fivefold cross-validation analysis, we additionally performed a Leave-One-Dataset-Out (LODO) evaluation on the CRC 16S cohorts. In each LODO iteration, one cohort was held out entirely as the test set, while the remaining CRC 16S cohorts were used for training. This cohort-level separation provides a direct assessment of model robustness across independent CRC cohorts.

### Network construction and analysis

To explore disease-associated ecological shifts, microbial co-occurrence networks were constructed using the ggClusterNet 2 package (v 2.0) (52) in R. To prevent confounding effects from inter-study batch variation inherent to multi-cohort data sets, this analysis was strictly performed on the T2D single-study metagenomic cohort (PRJNA422434). Profiles from control (*n* = 45) and T2D (*n* = 64) groups were analyzed independently to compare their topological architectures. Network edges were retained based on Spearman correlation coefficients (|*r*| > 0.6) and statistical significance (*P* < 0.05). Key network topological characteristics, including the number of nodes, positive/negative edges, and the average clustering coefficient, were calculated to quantify structural differences between health and disease states.

## Data Availability

All figure-generation scripts, processed data sets, model source code, metadata, and hyperparameter configurations are available at https://github.com/xu-research-lab/PhyloGCNE.

## SUPPLEMENTAL MATERIAL

Supplemental material aem.00788-26-s0001.docx Fig. S1 to S4; Tables S1 to S3.

ASM does not own the copyrights to Supplemental Material that may be linked to, or accessed through, an article. The authors have granted ASM a non-exclusive, world-wide license to publish the Supplemental Material files. Please contact the corresponding author directly for reuse.

## References

[1] Lloyd-Price J, Arze C, Ananthakrishnan AN, Schirmer M, Avila-Pacheco J, Poon TW, Andrews E, Ajami NJ, Bonham KS, Brislawn CJ, et al.. 2019. Multi-omics of the gut microbial ecosystem in inflammatory bowel diseases. Nature 569:655–662. doi:10.1038/s41586-019-1237-9 31142855 PMC6650278

[2] Wirbel J, Pyl PT, Kartal E, Zych K, Kashani A, Milanese A, Fleck JS, Voigt AY, Palleja A, Ponnudurai R, et al.. 2019. Meta-analysis of fecal metagenomes reveals global microbial signatures that are specific for colorectal cancer. Nat Med 25:679–689. doi:10.1038/s41591-019-0406-6 30936547 PMC7984229

[3] Gurung M, Li Z, You H, Rodrigues R, Jump DB, Morgun A, Shulzhenko N. 2020. Role of gut microbiota in type 2 diabetes pathophysiology. EBioMedicine 51:102590. doi:10.1016/j.ebiom.2019.11.051 31901868 PMC6948163

[4] Kovatcheva-Datchary P, Nilsson A, Akrami R, Lee YS, De Vadder F, Arora T, Hallen A, Martens E, Björck I, Bäckhed F. 2015. Dietary fiber-induced improvement in glucose metabolism is associated with increased abundance of *Prevotella*. Cell Metab 22:971–982. doi:10.1016/j.cmet.2015.10.001 26552345

[5] Shi C-L, Chen T, Lan C, Gan R-Y, Yu J, Zhao F, Liu Y-X. 2024. iMetaOmics: advancing human and environmental health through integrated meta-omics. IMetaOmics 1:e21. doi:10.1002/imo2.21 41675548 PMC12806273

[6] Knights D, Kuczynski J, Koren O, Ley RE, Field D, Knight R, DeSantis TZ, Kelley ST. 2011. Supervised classification of microbiota mitigates mislabeling errors. ISME J 5:570–573. doi:10.1038/ismej.2010.148 20927137 PMC3105748

[7] Marcos-Zambrano LJ, Karaduzovic-Hadziabdic K, Loncar Turukalo T, Przymus P, Trajkovik V, Aasmets O, Berland M, Gruca A, Hasic J, Hron K, et al.. 2021. Applications of machine learning in human microbiome studies: a review on feature selection, biomarker identification, disease prediction and treatment. Front Microbiol 12:634511. doi:10.3389/fmicb.2021.634511 33737920 PMC7962872

[8] Wang Q, Fan X, Wu S, Su X. 2024. PM-CNN: microbiome status recognition and disease detection model based on phylogeny and multi-path neural network. Bioinforma Adv 4:vbae013. doi:10.1093/bioadv/vbae013 PMC10873578 38371919

[9] Fioravanti D, Giarratano Y, Maggio V, Agostinelli C, Chierici M, Jurman G, Furlanello C. 2018. Phylogenetic convolutional neural networks in metagenomics. BMC Bioinformatics 19:49. doi:10.1186/s12859-018-2033-5 29536822 PMC5850953

[10] Wang B, Shen Y, Fang J, Su X, Xu ZZ. 2024. DeepPhylo: phylogeny‐aware microbial embeddings enhanced predictive accuracy in human microbiome data analysis. Advanced Science 11:2404277. doi:10.1002/advs.202404277 39403892 PMC11615782

[11] Zhang J, Meng F, Sun Y, Xu W, Wu S, Su X. 2025. Phylo-Spec: a phylogeny-fusion deep learning model advances microbiome status identification. mSystems 10:mSystems doi:10.1128/msystems.01453-25 PMC12710343 41283667

[12] Parada Venegas D, De la Fuente MK, Landskron G, González MJ, Quera R, Dijkstra G, Harmsen HJM, Faber KN, Hermoso MA. 2019. Short chain fatty acids (SCFAs)-mediated gut epithelial and immune regulation and its relevance for inflammatory bowel diseases. Front Immunol 10:277. doi:10.3389/fimmu.2019.00277 30915065 PMC6421268

[13] Png CW, Lindén SK, Gilshenan KS, Zoetendal EG, McSweeney CS, Sly LI, McGuckin MA, Florin THJ. 2010. Mucolytic bacteria with increased prevalence in IBD mucosa augment *in vitro* utilization of mucin by other bacteria. Off J Am Coll Gastroenterol ACG 105:2420–2428. doi:10.1038/ajg.2010.281 20648002

[14] Henke MT, Kenny DJ, Cassilly CD, Vlamakis H, Xavier RJ, Clardy J. 2019. *Ruminococcus gnavus*, a member of the human gut microbiome associated with Crohn’s disease, produces an inflammatory polysaccharide. Proc Natl Acad Sci USA 116:12672–12677. doi:10.1073/pnas.1904099116 31182571 PMC6601261

[15] Konikoff T, Gophna U. 2016. Oscillospira: a central, enigmatic component of the human gut microbiota. Trends Microbiol 24:523–524. doi:10.1016/j.tim.2016.02.015 26996766

[16] Valles-Colomer M, Falony G, Darzi Y, Tigchelaar EF, Wang J, Tito RY, Schiweck C, Kurilshikov A, Joossens M, Wijmenga C, et al.. 2019. The neuroactive potential of the human gut microbiota in quality of life and depression. Nat Microbiol 4:623–632. doi:10.1038/s41564-018-0337-x 30718848

[17] Moon C, Baldridge MT, Wallace MA, D C-A, Virgin HW, Stappenbeck TS. 2015. Vertically transmitted faecal IgA levels determine extra-chromosomal phenotypic variation. Nature 521:90–93. doi:10.1038/nature14139 25686606 PMC4425643

[18] Chen J, Wright K, Davis JM, Jeraldo P, Marietta EV, Murray J, Nelson H, Matteson EL, Taneja V. 2016. An expansion of rare lineage intestinal microbes characterizes rheumatoid arthritis. Genome Med 8:43. doi:10.1186/s13073-016-0299-7 27102666 PMC4840970

[19] Desai MS, Seekatz AM, Koropatkin NM, Kamada N, Hickey CA, Wolter M, Pudlo NA, Kitamoto S, Terrapon N, Muller A, et al.. 2016. A dietary fiber-deprived gut microbiota degrades the colonic mucus barrier and enhances pathogen susceptibility. Cell 167:1339–1353. doi:10.1016/j.cell.2016.10.043 27863247 PMC5131798

[20] Everard A, Belzer C, Geurts L, Ouwerkerk JP, Druart C, Bindels LB, Guiot Y, Derrien M, Muccioli GG, Delzenne NM, et al.. 2013. Cross-talk between *Akkermansia muciniphila* and intestinal epithelium controls diet-induced obesity. Proc Natl Acad Sci USA 110:9066–9071. doi:10.1073/pnas.1219451110 23671105 PMC3670398

[21] Holscher HD. 2017. Dietary fiber and prebiotics and the gastrointestinal microbiota. Gut Microbes 8:172–184. doi:10.1080/19490976.2017.1290756 28165863 PMC5390821

[22] Qin J, Li Y, Cai Z, Li S, Zhu J, Zhang F, Liang S, Zhang W, Guan Y, Shen D, et al.. 2012. A metagenome-wide association study of gut microbiota in type 2 diabetes. Nature 490:55–60. doi:10.1038/nature11450 23023125

[23] Kalkan AT, Yorulmaz G, Akalin A, Dinleyici EC. 2025. Intestinal microbiota composition in patients with type 2 diabetes and effects of oral antidiabetics. J Clin Med 14:2786. doi:10.3390/jcm14082786 40283615 PMC12027695

[24] Korpela K, Salonen A, Virta LJ, Kekkonen RA, Forslund K, Bork P, de Vos WM. 2016. Intestinal microbiome is related to lifetime antibiotic use in Finnish pre-school children. Nat Commun 7:10410. doi:10.1038/ncomms10410 26811868 PMC4737757

[25] Pushalkar S, Ji X, Li Y, Estilo C, Yegnanarayana R, Singh B, Li X, Saxena D. 2012. Comparison of oral microbiota in tumor and non-tumor tissues of patients with oral squamous cell carcinoma. BMC Microbiol 12:144. doi:10.1186/1471-2180-12-144 22817758 PMC3507910

[26] Ferreira RM, Pereira-Marques J, Pinto-Ribeiro I, Costa JL, Carneiro F, Machado JC, Figueiredo C. 2018. Gastric microbial community profiling reveals a dysbiotic cancer-associated microbiota. Gut 67:226–236. doi:10.1136/gutjnl-2017-314205 29102920 PMC5868293

[27] Erawijantari PP, Mizutani S, Shiroma H, Shiba S, Nakajima T, Sakamoto T, Saito Y, Fukuda S, Yachida S, Yamada T. 2020. Influence of gastrectomy for gastric cancer treatment on faecal microbiome and metabolome profiles. Gut 69:1404–1415. doi:10.1136/gutjnl-2019-319188 31953253 PMC7398469

[28] Oh M, Zhang L. 2020. DeepMicro: deep representation learning for disease prediction based on microbiome data. Sci Rep 10:6026. doi:10.1038/s41598-020-63159-5 32265477 PMC7138789

[29] Lee SJ, Rho M. 2022. Multimodal deep learning applied to classify healthy and disease states of human microbiome. Sci Rep 12:824. doi:10.1038/s41598-022-04773-3 35039534 PMC8763943

[30] Martiny JBH, Jones SE, Lennon JT, Martiny AC. 2015. Microbiomes in light of traits: a phylogenetic perspective. Science 350:aac9323. doi:10.1126/science.aac9323 26542581

[31] Bichat A, Plassais J, Ambroise C, Mariadassou M. 2020. Incorporating phylogenetic information in microbiome differential abundance studies has no effect on detection power and FDR control. Front Microbiol 11:649. doi:10.3389/fmicb.2020.00649 32351481 PMC7174607

[32] Washburne AD, Silverman JD, Leff JW, Bennett DJ, Darcy JL, Mukherjee S, Fierer N, David LA. 2017. Phylogenetic factorization of compositional data yields lineage-level associations in microbiome datasets. PeerJ 5:e2969. doi:10.7717/peerj.2969 28289558 PMC5345826

[33] Van Calster B, Wynants L. 2019. Machine learning in medicine. N Engl J Med 380:2588. doi:10.1056/NEJMc1906060 31242379

[34] Barredo Arrieta A, Díaz-Rodríguez N, Del Ser J, Bennetot A, Tabik S, Barbado A, Garcia S, Gil-Lopez S, Molina D, Benjamins R, et al.. 2020. Explainable artificial intelligence (XAI): concepts, taxonomies, opportunities and challenges toward responsible AI. Information Fusion 58:82–115. doi:10.1016/j.inffus.2019.12.012

[35] Bi D, Zhu Y, Gao Y, Li H, Zhu X, Wei R, Xie R, Cai C, Wei Q, Qin H. 2022. Profiling *Fusobacterium* infection at high taxonomic resolution reveals lineage-specific correlations in colorectal cancer. Nat Commun 13:3336. doi:10.1038/s41467-022-30957-6 35680952 PMC9184491

[36] Zepeda-Rivera M, Minot SS, Bouzek H, Wu H, Blanco-Míguez A, Manghi P, Jones DS, LaCourse KD, Wu Y, McMahon EF, et al.. 2024. A distinct *Fusobacterium nucleatum* clade dominates the colorectal cancer niche. Nature 628:424–432. doi:10.1038/s41586-024-07182-w 38509359 PMC11006615

[37] Kang C, Lee J, Baek M-G, Kim N-E, Son H, Yoo S, Yi H. 2025. Urinary microbiome in non-muscle invasive bladder cancer: impact of sample types and sex differences. BMC Microbiol 25:623. doi:10.1186/s12866-025-04367-9 41039241 PMC12492864

[38] Martins EP, Hansen TF. 1997. Phylogenies and the comparative method: a general approach to incorporating phylogenetic information into the analysis of interspecific data. Am Nat 149:646–667. doi:10.1086/286013

[39] Jiang B, Zhang Z, Lin D, Tang J, Luo B. 2019. Semi-supervised learning with graph learning-convolutional networks. 2019 IEEE/CVF Conference on Computer Vision and Pattern Recognition (CVPR); Long Beach, CA, USA: , p 11313–11320. doi:10.1109/CVPR.2019.01157

[40] Luo Y, Shi L, Wu X-M. 2025. Can classic GNNs be strong baselines for graph-level tasks? Simple architectures meet excellence. ArXiv

[41] Smilkov D, Thorat N, Kim B, Viégas F, Wattenberg M. 2017. Smoothgrad: removing noise by adding noise. ArXiv

[42] Pedregosa F, Varoquaux G, Gramfort A, Michel V, Thirion B, Grisel O, Blondel M, Prettenhofer P, Weiss R, Dubourg V. 2011. Scikit-learn: machine learning in python. J Mach Learn Res 12:2825–2830.

[43] Callahan BJ, McMurdie PJ, Rosen MJ, Han AW, Johnson AJA, Holmes SP. 2016. DADA2: high-resolution sample inference from illumina amplicon data. Nat Methods advance online publication. doi:10.1101/024034 PMC4927377 27214047

[44] McDonald D, Jiang Y, Balaban M, Cantrell K, Zhu Q, Gonzalez A, Morton JT, Nicolaou G, Parks DH, Karst SM, et al.. 2024. Greengenes2 unifies microbial data in a single reference tree. Nat Biotechnol 42:715–718. doi:10.1038/s41587-023-01845-1 37500913 PMC10818020

[45] Bolyen E, Rideout JR, Dillon MR, Bokulich NA, Abnet CC, Al-Ghalith GA, Alexander H, Alm EJ, Arumugam M, Asnicar F, et al.. 2019. Reproducible, interactive, scalable and extensible microbiome data science using QIIME 2. Nat Biotechnol 37:852–857. doi:10.1038/s41587-019-0209-9 31341288 PMC7015180

[46] Han Z, Hu Y, Lin X, Cheng H, Dong B, Liu X, Wu B, Xu ZZ. 2025. Systematic analyses uncover robust salivary microbial signatures and host-microbiome perturbations in oral squamous cell carcinoma. mSystems 10:e01247–24. doi:10.1128/msystems.01247-24 PMC11834404 39873508

[47] Li Y, Hu Y, Zhan X, Song Y, Xu M, Wang S, Huang X, Xu ZZ. 2023. Meta-analysis reveals *Helicobacter pylori* mutual exclusivity and reproducible gastric microbiome alterations during gastric carcinoma progression. Gut Microbes 15:2197835. doi:10.1080/19490976.2023.2197835 37020297 PMC10078126

[48] Nadeau C, Bengio Y. 1999. Inference for the generalization error. *In* Advances in neural information processing systems. Vol. 12.

[49] Bouckaert RR, Frank E. 2004. Pacific-Asia conference on knowledge discovery and data mining, p 3–12. *In* Evaluating the replicability of significance tests for comparing learning algorithms

[50] Henderson T. 2023. correctR: corrected test statistics for comparing machine learning models on correlated samples. R package version 0.1.3. https://hendersontrent.github.io/correctR.

[51] McMurdie PJ, Holmes S. 2013. Phyloseq: an R package for reproducible interactive analysis and graphics of microbiome census data. PLoS One 8:e61217. doi:10.1371/journal.pone.0061217 23630581 PMC3632530

[52] Wen T, Liu Y, Liu L, Niu G, Ding Z, Teng X, Ma J, Liu Y, Yang S, Xie P, et al.. 2025. ggClusterNet 2: an R package for microbial co‐occurrence networks and associated indicator correlation patterns. iMeta 4:e70041. doi:10.1002/imt2.70041 40469522 PMC12130565

