# Supplementary material for: Adaptive graph learning of microbial phylogeny enables accurate and interpretable microbiome-based host phenotype prediction
Source: Appl Environ Microbiol. 2026 Jul 10;92(8):e00788-26. doi: 10.1128/aem.00788-26 (PMC13488359; doi:10.1128/aem.00788-26)
Supplement: Supplementary file 1 [file aem.00788-26-s0001.docx]

**Supplemental Material**

Supplementary Figure S1. Confusion matrices for the multiclass classification task. Heatmaps show per-class prediction results for the five diagnostic categories, including Control, CRC, IBD, IBS, and ASD. Rows indicate true labels, columns indicate predicted labels, and cell values represent the proportion of samples assigned to each predicted class, as indicated in the color scale. Red dashed boxes highlight the healthy Control group. Phylo_Spec and RF achieve high disease recall (sensitivity) at the expense of misclassifying Control samples, leading to high false-positive rates, PhyloGCNE demonstrates balanced predictions. PhyloGCNE correctly classifies 72% of healthy controls while maintaining competitive Macro-F1 (0.66) and Macro-Precision (0.64) scores.

Supplementary Figure S2. Leave-One-Dataset-Out (LODO) evaluation on the CRC 16S cohorts. In each iteration, one CRC 16S cohort was held out entirely as the external test set, while the remaining CRC 16S cohorts were used for model training. Each point represents the AUC obtained on one held-out cohort. This analysis evaluates model robustness across independent CRC cohorts.

Supplementary Figure S3. Schematic definition of the full-tree and flat-structure graph settings used in the ablation analysis. In the full-tree setting, observed taxa are represented as leaf nodes, internal ancestral nodes are retained, and edges follow the original phylogenetic topology with branch-length-derived edge attributes. In the flat-structure setting, internal nodes are removed and all observed leaf taxa are connected directly to a single root node, forming a star topology. Leaf abundances are kept unchanged, all flat-structure branch lengths are set uniformly to 1, and the same GNN architecture, model hyperparameters, and training protocol are used as in the full-tree setting.

Supplementary Figure S4. Co-occurrence networks for the control and T2D groups.

Microbial co-occurrence networks were constructed independently for the control (left) and T2D (right) groups using ggClusterNet 2 (PRJNA422434; control: n = 45; T2D: n = 64). Edges represent significant pairwise Spearman correlations (|r| > 0.6, *p* < 0.05), with orange and blue edges indicating positive and negative correlations, respectively. Node color denotes phylum-level taxonomy; node size is proportional to degree.

Supplementary Table S1 Information of microbiome datasets

| Dataset | Disease/Task | Seq. Type | Accession Numbers | Sample size | Status | No. of microbes |
| --- | --- | --- | --- | --- | --- | --- |
| Synthetic dataset | Phylogenetic signal validation | Simulation | NA |  | Group S1 + S3, *n* = 77  Group S2 + S4, n = 71 | 60 |
| IBD 16S | Inflammatory Bowel Disease | 16S rRNA gene amplicon | PRJNA418765  PRJNA450340 PRJNA317429 PRJEB13679 PRJEB13680 PRJEB42155 PRJEB23009 PRJNA313074 | Ctrl/IBD: 0/796  Ctrl/IBD: 20/39  Ctrl/IBD: 33/11  Ctrl/IBD: 220/359  Ctrl/IBD: 14/111  Ctrl/IBD: 5/75  Ctrl/IBD: 104/204  Ctrl/IBD: 10/9 | Control, *n* = 406  IBD, n = 1604 | 416 |
| CRC 16S | Colorectal Cancer | 16S rRNA gene amplicon | PRJEB6070  PRJNA280026  PRJNA911189  PRJNA325650  PRJNA290926  PRJNA318004 | Ctrl/CRC: 55/32  Ctrl/CRC: 14/7  Ctrl/CRC: 40/30  Ctrl/CRC: 6/17  Ctrl/CRC: 84/33  Ctrl/CRC: 69/25 | Control, *n* = 268  CRC, n = 144 | 1,495 |
| Fiber 16S | Dietary fiber response | 16S rRNA gene amplicon | PRJEB41443 PRJNA293971 PRJNA306884  PRJNA891951 PRJNA428736 PRJNA560950 PRJNA780023 | Ctrl/Fiber: 89/29  Ctrl/Fiber: 40/20  Ctrl/Fiber: 78/75  Ctrl/Fiber: 152/166  Ctrl/Fiber: 708/468  Ctrl/Fiber: 79/118  Ctrl/Fiber: 148/100 | Control, *n* = 1,294  Fiber, n = 976 | 930 |
| OSCC 16S | Oral Squamous Cell Carcinoma | 16S rRNA gene amplicon | PRJEB39064 OEP000837 PRJNA744870 PRJNA751046 PRJNA756784 PRJNA386665 PRJNA421234 | Ctrl/OSCC: 25/27  Ctrl/OSCC: 12/32  Ctrl/OSCC: 76/77  Ctrl/OSCC: 18/9  Ctrl/OSCC: 39/20  Ctrl/OSCC: 107/105  Ctrl/OSCC: 16/13 | Control, n = 293  OSCC, n = 283 | 1,162 |
| GC 16S | Gastric cancer | 16S rRNA gene amplicon | PRJEB21104 PRJEB21497 PRJEB26931 PRJNA375772 PRJNA481413 | G/GC: 43/10  G/GC: 3/12  G/GC: 38/156  G/GC: 223/73  G/GC: 115/110 | Gastritis, n = 422  GC, n = 361 | 2,229 |
| CRC WGS | Colorectal Cancer | Metagenome | ERP005534 SRP136711 PRJNA397112 PRJNA531273 PRJEB7774 | Ctrl/CRC: 15/20  Ctrl/CRC: 21/28  Ctrl/CRC: 22/0  Ctrl/CRC: 0/9  Ctrl/CRC: 9/17 | Control, n = 67  CRC, n = 74 | 354 |
| T2D WGS | Type 2 Diabetes | Metagenome | PRJNA422434 | Ctrl/T2D: 45/64 | Control, n = 45  T2D, n = 64 | 420 |
| Multiclass | IBD + CRC + IBS + ASD | 16S rRNA gene amplicon | IBD 16S  CRC 16S PRJNA544721 PRJNA282013 PRJNA578223 PRJNA589343 | See rows above  See rows above  Ctrl/IBS: 31/84  Ctrl/ASD: 22/59  Ctrl/ASD: 38/2  Ctrl/ASD: 34/77 | Control, *n* = 704  CRC, n = 142  IBD, n = 914  IBS, n = 84  ASD, n = 138 | 585 |

Note: For the multiclass task, IBD and CRC samples were derived as subsets from the IBD 16S and CRC 16S cohorts listed above. Therefore, the accession-level sample sizes shown for the binary IBD and CRC benchmarks should not be interpreted as the exact sample composition of the multiclass task.

Supplementary Table S2. Hyperparameter grid search space and final optimal configurations for all compared models, determined on the in silico synthetic dataset.

| Model | Hyperparameter | Search space (Grid) | Optimal value |
| --- | --- | --- | --- |
| PhyloGCNE | Learning Rate (AdamW) | { 1×10^-4^, 5×10^-4^, 1×10^-3^} | 1 × 10^-3^ |
|  | Batch Size | { 8, 16, 32, 64 } | 32 |
|  | Hidden Dimension | { 32, 64, 100, 128 } | 100 |
|  | GCNE blocks | { 2, 4, 6 } | 4 |
|  | Weight Decay | { 0.0, 1×10^-5^, 1×10^-4^ } | 1×10^-5^ |
| Phylo-Spec | Learning Rate | { 1×10⁻^4^, 5×10^-4^, 1×10^-3^} | 1 × 10^-4^ |
|  | Batch Size | { 16, 32, 64 } | 64 |
|  | Hidden size | { 32, 64, 100, 128 } | 32 |
|  | Kernel size | { 3, 5, …, 15} | 3 |
| DeepPhylo | Learning Rate | { 1×10^-4^, 5×10^-4^, 1×10^-3^} | 1 × 10^-4^ |
|  | Batch Size | { 32, 64, 128 } | 64 |
|  | Hidden size | { 32, 64, 128 } | 32 |
|  | Kernel size | {3, 5, …, 15} | 7 |
| Random Forest | Number of Trees (n_estimators) | { 100, 300, 500, 1000 } | 500 |
|  | Maximum Depth (max_depth) | { 4, 5, None } | 5 |
|  | Min Samples Split | { 2, 5, 10} | 2 |

Supplementary Table S3. Topological properties for the control and T2D groups

| Topological properties | Control | T2D |
| --- | --- | --- |
| vertices | 42 | 30 |
| edges | 61 | 36 |
| neg.edges | 9 | 0 |
| Average CC | 0.448 | 0.206 |
